# Pyloric gland adenoma with low-grade intraepithelial neoplasia: A case report and literature review

**DOI:** 10.1097/MD.0000000000026378

**Published:** 2021-06-25

**Authors:** Hai-Long Li, Yan Wang, Yu-Bo Ren, Xue-Song Yang, Li Wang, Lei Zhang, Xiang-Chun Lin

**Affiliations:** a Department of Gastroenterology; b Department of Pathology, Peking University International Hospital, China.

**Keywords:** case report, endoscopic morphology, pathological features, pyloric gland adenoma

## Abstract

**Rationale:**

Pyloric gland adenoma (PGA) is often associated with pyloric gland metaplasia. It has high malignant potential but a low clinical diagnosis rate. Therefore, we reported a case of PGA and reviewed the literature to summarize the clinicopathological features of pyloric adenoma.

**Patient concerns:**

A 62-year-old female underwent gastroscopy due to intermittent acid regurgitation and heartburn, which revealed a 4×6 mm flat, elevated lesion in the greater curvature of the upper gastric body, with depression in the central region and blood scab attachment.

**Diagnosis and intervention:**

Biopsy revealed gastric adenoma with low-grade intraepithelial neoplasia. The patient was treated with ESD, and pathology showed gastric pyloric gland adenoma with low-grade dysplasia. The cells were positive for MUC6 and MUC5AC immunohistochemically.

**Outcomes:**

The patient received proton pump inhibitors and gastric mucosal protective agents for one month after ESD. She occasionally presented acid regurgitation and heartburn, with no abdominal pain, abdominal distension, melena, or hematochezia. Follow-up gastroscopy will be reexamined 1 year later.

**Lessons:**

PGA has nonspecific performance under endoscopy, and its diagnosis mainly depends on pathology. Clinicians need to increase their ability to recognize such lesions and treat them in time to improve the prognosis.

## 1 Introduction

Pyloric gland adenoma is a type of gastric adenoma.^[1]^ It has attracted increasing attention in recent years, and its incidence is low, accounting for 2–2.7% of gastric adenomas. Pyloric gland adenoma is a precancerous lesion that can evolve into adenocarcinoma through low-grade intraepithelial neoplasia to high-grade intraepithelial neoplasia, with a reported carcinogenesis rate of 12–47%.^[2–4]^ However, PGA has no specific symptoms, and anemia related to autoimmune gastritis (AIG) is more common.^[5]^

Therefore, it is necessary for clinicians to be aware of this entity. We report a case of pyloric gland adenoma found by routine gastroscopy and summarize the endoscopic and pathological features of PGA through a literature review.

## 2 Case report

A 62-year-old woman suffered intermittent acid regurgitation and heartburn for 3 years. She occasionally had dull pain in the upper abdomen but no nausea, vomiting, melena or weight loss. She took omeprazole irregularly to reduce the symptoms. She had a history of chronic superficial gastritis and *Helicobacter pylori* infection, which was eradicated successfully 2 years ago. Her father died of pancreatic cancer and had no family history of gastric or colon cancer. There were no abnormalities during physical examination.

**Figure 1:**
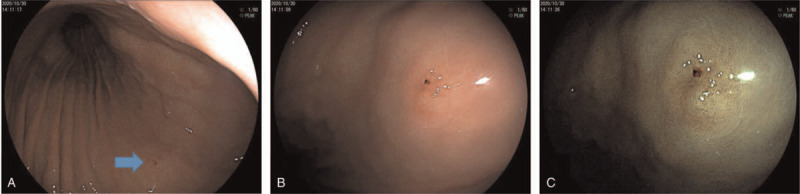
(A) The lesion was located in the great curvature of the upper segment of the gastric body, as shown by the arrow. (B) Observation showed that the lesion was type IIa, colorless change, old blood could be seen at the central depression, and the lesion size was 4×6 mm in size. In FICE mode image (C), the demarcation line of the lesion was positive, and the surface pattern was dense and slightly disordered.

**Figure 2:**
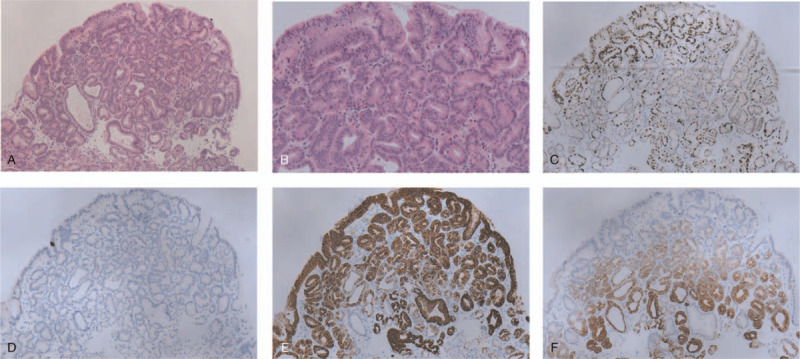
Pathological images showing PGA with low-grade intraepithelial neoplasia (A) (100×); (B) (200×). HE staining showed dense tubular glands covered with monolayer cuboidal to low columnar epithelial cells, mild structural disorder, slightly elongated nuclei, no obvious nucleoli, light staining to eosinophilic cytoplasm, ground glass shape, and no apical mucinous cap. (C–F) Immunohistochemistry. (C) (100×) Ki67 positivity of less than 1%. (D) (100×) MUC2 negative. (E) (100×) MUC5AC positive. (F) (100×) MUC6 positive.

Gastroscopy revealed a type IIa lesion in the greater curvature of the upper gastric body, 4×6 mm in size, colorless change, that exhibited depression in its center and blood scab attachment. Close observation and FICE mode image enhancement showed that the demarcation line was positive and the surface pattern was slightly disordered (Fig. 1). The pathology of the biopsy showed gastric adenoma with low-grade intraepithelial neoplasia. Immunohistochemical staining revealed MUC6 (+), MUC5ac (+), MUC2 (-), CD10 (-), Syn (+), CgA (+), p53 (mutant), Ki67 (+), Pep-I (-), and ATPase (-) (Fig. 2).

The patient was treated with endoscopic submucosal dissection (ESD). The pathological results showed that resected mucosal tissue was 4×3.3 cm in size, and there was a superficial protuberant lesion (Type 0–IIa) on the surface of approximately 0.6×0.4 cm. The histological diagnosis was gastric adenoma of the pyloric type (with low-grade dysplasia), the lesion was limited to the mucosal layer, and the horizontal and vertical margins were negative. Immunohistochemistry staining showed MUC6 (partial +), MUC5AC (partial +), MUC2 (–), CD10 (–), Syn (–), p53 (wild type), Ki-67 (less than 5%), Pepsinogen-I (–), Pepsinogen-II (partial +), and ATPase (–).

The patient recovered well and was followed up for 6 months. At present, she has no abdominal discomfort. Gastroscopy was scheduled to be reexamined 12 months after the operation.

## 3 Discussion

Gastric cancer is a common tumor of the digestive system, and it has always been a hot topic for researchers. The occurrence of traditional gastric adenocarcinoma is the result of the stepwise progression of gastric mucosal inflammation (*Helicobacter pylori* infection-atrophy and intestinal metaplasia-dysplasia-gastric cancer).^[6,7]^ However, several studies have demonstrated well-differentiated adenocarcinomas arising in nonintestinalized gastric mucosa, such as gastric-type well-differentiated adenocarcinomas in hyperplastic polyps of the stomach in 1985. There has been a question of whether well-differentiated adenocarcinomas of the stomach are exclusively intestinal type. Along with the development of research, gastric-type well-differentiated adenocarcinoma has been proposed.^[8]^ According to the WHO classification standard published in 2019, gastric adenomas are divided into intestinal type and gastric type according to the direction of differentiation, while gastric type adenomas are further divided into pyloric gland type and foveolar type.^[9]^ In Japan, gastric adenoma refers to pyloric gland adenoma, while foveolar-type intraepithelial neoplasia/adenoma is classified as highly differentiated adenocarcinoma.

PGA was first reported by Elster in 1976.^[10]^ The incidence of pyloric gland adenoma is relatively low. It occurs more frequently in older women (female:male ratio of 3:1) and often exists in the form of polyps in the stomach (69%).^[11,12,13]^ It can also occur in the duodenum, esophagus (Barrett's esophagus), bile duct, gallbladder, pancreas, small intestine, rectum and even cervix,^[5]^ mostly related to pyloric glandular metaplasia.^[4]^ Histologically, PGA consists of dense tubular glands (occasionally cystic dilatation). The lesions were covered with monolayer cuboidal to low columnar epithelial cells with round nuclei at the base of the cells, no obvious nucleoli, and lightly stained eosinophilic cytoplasm, showing a ground glass shape and no apical mucinous cap. Immunohistochemically, the tumor cells were positive for MUC6 and MUC5AC and negative for intestinal-type markers such as MUC2, CD10 and CDX2. It has been reported that MUC6 and MUC5AC have unique immunohistochemical expression patterns useful for the diagnosis of PGA. MUC6 is diffusely and strongly positive in the whole PGA lesion, while MUC5AC has low or no (lost) expression in the surface epithelium. Ekrem Çakar et al reported a case of PGA with high-grade dysplasia, which suggests that PGA has the potential to become cancerous, which requires the attention of clinicians and pathologists.

PGA has different performance under endoscopy and is difficult to diagnose accurately. Therefore, we reviewed the relevant literature and summarized the endoscopic manifestations, pathological features, and gene and chromosome mutations associated with PGA (Table 1) to improve the understanding of the disease by clinicians and pathologists.

**Table 1:** The endoscopic and pathologic characteristics of pyloric gland adenoma reported in the literature and the case report.

Author/year	Cases	Age/Sex	Site	Endoscopic morphology	Size	Status of the unaffected gastric mucosa	Magnifying endoscope/EUS	Immunoreactivity	Carcinogenesis rate
Vieth et al^[14]^/2003	90	73/ F:M 3:1	Corpus 58 Cardia 7 Antrum 6 Intermediate zone 4	–	7–25.2 mm	HP+ 16 HP eradication 6 AIG 18 Normal 2	–	–	30%
Golger et al^[15]^/2008	1	79/F	Antrum	Polyp	20 mm	HP-	IMVP-asteroid-shaped mucosal pits	MUC6+	–
Chen et al^[2]^/2009	41	73/ F:M 25:11	Body of stomach 9 antrum 1	–	–	intestinal metaplasia 6 AIG 4	–	MUC6+ MUC5AC+ MUC2− CDX2−	–
Çakar et al^[12]^/2013	1	60/M	proximal gastric corpus	polyp with a lobulated surface	20 mm	–	–	MUC6+ MUC5AC+	–
Salem SB et al^[16]^/2014	1	74/M	fundus	polypoid lesion	20 mm	–	DL+ IMVP+IMSP+ granular surface structure	MUC6+ MUC5AC+	
Nakajo et al ^[1]^/2018	1	80/F	greater curvature of the middle gastric body	flat elevated lesion	20 mm	HP eradication	IMVP+ including closed-loop vessels with repeated irregular anastomoses	MUC6+ MUC5AC+	–
Choi et al^[2]^/2018	67	66/ F:M 30:27	body/fundus 45 cardia 7 antrum 5 gastroesophageal junction 4 pylorus 2	polypoid lesion or mass 62 mucosal irregularity 2 flat lesion 1 ulcer 1	–	AIG 15 Normal 24 HP+ 2 atrophic gastritis2	–	MUC6+ MUC5AC+	16.4%
Pei et al^[17]^/2019	1	75/M	cardia	flat, elevated lesion	20 mm	–	IMVP+	MUC6+ MUC5AC+	–
Min et al^[11]^/2020	1	69/M	posterior wall of the upper part of the gastric body	SMT-like elevated lesion, with an opening on the surface of the tumor	10 mm	HP-nonatrophic gastritis	IMVP+, IMSP+ the orifice showed dilated glandular duct/EUS: equal echoic mass with several cysts located in the submucosal layer with an intact muscularis	MUC6+ MUC5AC+ MUC4- P53- Ki67 2%	–
Present case	1	62/F	Greater curvature of upper gastric body	flat, elevated lesion	6 mm	HP-nonatrophic gastritis	–	MUC6+ MUC5AC+ MUC2-	–

AIG = autoimmune gastritis, DL = demarcation line, EUS = endoscopic ultrasound, HP = *Helicobacter pylori*, IMSP = irregular microsurface pattern, IMVP = irregular microvascular pattern, SMT = submucosal tumor.

Stomach PGA was mostly found in the fundus and body (64%), followed by the cardia (8%) and antrum (7%) of the stomach; the intermediate zone is the least common (5%).^[14]^ There have also been a few cases in the remnant stomach. Under white-light model gastroscopy, most PGA lesions appear as polypoid and nodular protuberances or as uneven mucosa, flat eminences, ulcer-like lesions or submucosal tumor (SMT)-like lesions.^[2,12,18]^ The average lesion size is 1.0 –2.5 cm. Some cases suggest that there are openings on the surface of the protuberant lesions, which should be distinguished from ectopic pancreas, neuroendocrine tumors (NETs), SMT-like adenocarcinoma and proliferative polyps. Immunohistochemistry is helpful for differential diagnosis and for judging the source of lesions of nonmucosal origin.^[4]^ The relationship between PGA and background mucosa is still controversial. Vieth M et al investigated 90 lesions of PGA and showed that autoimmune gastritis (AIG) was found in 34% of cases, of which the infection rate of HP was 30%. Only 3.8% of cases had a normal gastric mucosa.^[14]^ However, the results of another study were quite the opposite: 22.4% of PGA patients had AIG, and 35.8% had normal gastric mucosa.^[2]^ Studies by Zong-Ming Chen et al showed that 60% of PGA showed an intestinal metaplasia background, and 40% of lesions were associated with AIG.^[13]^ Thus, further study is needed to clarify the relationship between PGA and background mucosa. At present, there is a lack of data about the manifestations of PGA under magnifying endoscopy and endoscopic ultrasound. According to the literature reports, the surface pattern of the lesion is star-shaped or elongated in magnified NBI mode. Demarcation lines, surface microvasculature (loop-like vessels) and surface microstructure pattern changes, and even structural loss, will be observed when accompanied by intraepithelial neoplasia or even carcinogenesis. The lesions were located in the superficial layer of the mucosa; even in the submucosa, there were isoechoic patterns, and some areas showed nonechoic patterns under endoscopic ultrasonography.^[11,16]^

According to the immunohistochemical characteristics, PGA can be divided into three types:

1. mixed type: both MUC6 and MUC5AC are expressed, but MUC6 expression is much greater, and the expression of MUC6 in deep glands is generally between 20% and 90%;
2. pure pyloric type: MUC6 is diffusely expressed, and MUC5AC is expressed only in the superficial fovea epithelium; and
3. foveolar-dominant type: diffuse MUC5AC expression, MUC6 expression in deep glands < 10%. Mixed PGA is more common.^[2]^

In the early stage of PGA, there may be no change in dysplasia; rather, this stage is mainly characterized by the dense distribution of glands. After the appearance of dysplasia, the nuclear/cytoplasmic ratio is increased. It has been reported that the expression of p53 in PGA is lower than that in intestinal adenoma but increases progressively from high-grade intraepithelial neoplasia to adenocarcinoma in PGA. The high expression of p53 may indicate that PGA has a relatively high carcinogenic potential.^[19]^ A Japanese study showed that the loss rate of mismatch gene repair proteins in PGA was similar to that in intestinal adenomas but much lower than that in foveolar-type adenomas, suggesting that PGA microsatellites are relatively stable. Namrata Setia et al confirmed this through second-generation sequencing.^[20]^ However, the results of another US study were contradictory, and further study according to ethnicity is needed.^[5]^

Kushima et al successfully confirmed the instability and precancerous nature of PGA for the first time and confirmed the “pyloric adenoma-adenocarcinoma sequence” of PGA by comparative genomic hybridization (CGH) analysis, which showed that PGA shared genetic and phenotypic characteristics with gastric adenocarcinoma.^[9]^ GNAS and KRAS mutations were also found to occur frequently in gastric PGA. Namrata Setia et al showed that the mutation rates of GNAS and KRAS in PGA were 67% and 41%, respectively, and that 2 mutations existed simultaneously in most cases.^[20]^ Histological techniques and CGH analysis showed amplifications of chromosomes 17pq and 20q in PGA and amplification of chromosome 20q in invasive gastric adenomas. Thus, the above studies on the genetic and chromosomal features of PGA confirm its high carcinogenic potential.

It has been reported in the literature that larger lesions, villous tubular structure, AIG and mixed PGA are more likely to become cancerous. Although the local recurrence rate of PGA is less than 10%, it is necessary to follow up according to the postoperative pathological results.^[2]^

The patient's lesions were Type 0-IIa, located on the greater curvature of the upper part of the stomach. Pathology showed that the glands and cells were similar to the pyloric glands and were accompanied by low-grade intraepithelial neoplasia. Immunohistochemistry showed that MUC5AC was diffusely expressed, and deep glands expressed MUC6, which meets foveolar-dominant type PGA.

In the process of endoscopy, endoscopists should pay attention to whether there is the possibility of PGA when finding protuberant lesions of the stomach body, which need to be differentiated from fundus glandular gastric cancer, hyperplastic polyps, and submucosal tumors. Detailed observation, biopsy and pathological examination, especially immunohistochemistry, play an important role in its diagnosis. To improve the diagnostic ability of such diseases. First, endoscopists were needed to learn more about PGA, including predilection sites, endoscopic morphological characteristics and high-risk groups. Second, endoscopists should strictly follow the endoscopic examination standard specifications and carefully examine during the operation. Finally, endoscopists and pathologists were needed to maintain good communication. For the disease to be diagnosed, endoscopists should give the pathologist a reminder, and the final diagnosis should be given after mutual consultation.

## 4 Conclusion

Pyloric gland adenoma is common in elderly women. Under endoscopy, most of the lesions were polypoid-like lesions, which usually occurred in the fundus and body of the stomach. The pyloric gland-like structure was observed pathologically under light microscopy. Immunohistochemistry, MUC6 and MUC5AC were helpful for diagnosis. Because of its malignant potential, clinicians should attend to the possibility of pyloric gland adenoma and pursue timely treatment.

## Author contributions

**Conceptualization:** Hai-Long Li, Yan Wang, Xue-Song Yang, Li Wang, Lei Zhang, xiangchun Lin.

**Resources:** Yu-Bo Ren.

**Writing – original draft:** Hai-Long Li, Yan Wang.

**Writing – review & editing:** xiangchun Lin.

## References

[1] Nakajo K Oono Y Kuwata T. Case of pyloric gland adenoma accompanied by a component of foveolar epithelial-type adenoma within the lesion. Dig Endosc 2018;30:673. 29729058 10.1111/den.13180

[2] Choi W-T Brown I Ushiku T. Gastric pyloric gland adenoma: a multicenter clinicopathological study of 67 cases. Histopathology 2018;72:1007–14. 29278427 10.1111/his.13460

[3] Chen Z-M Scudiere JR Abraham SC Montgomery E. Pyloric gland adenoma: an entity distinct from gastric foveolar type adenoma. Am J Surg Pathol 2009;33:186–93. 18830123 10.1097/PAS.0b013e31817d7ff4

[4] Vieth M Montgomery EA. Some observations on pyloric gland adenoma: an uncommon and long ignored entity!. J Clin Pathol 2014;67:883–90. 25092673 10.1136/jclinpath-2014-202553

[5] Pezhouh MK Y J. Gastric pyloric gland adenoma. Arch Pathol Lab Med 2015;139:823–6. 26030253 10.5858/arpa.2013-0613-RS

[6] Torre LA Bray F Siegel RL Ferlay J Lortet-Tieulent J Jemal A. Global cancer statistics, 2012. CA Cancer J Clin 2015;65:87–108. 25651787 10.3322/caac.21262

[7] Dinis-Ribeiro M Areia M de Vries A. Management of precancerous conditions and lesions in the stomach (MAPS): guideline from the European Society of Gastrointestinal Endoscopy (ESGE), European Helicobacter Study Group (EHSG), European Society of Pathology (ESP), and the Sociedade Portuguesa de Endoscopia Digestiva (SPED). Endoscopy 2011;44:74–94. 22198778 10.1055/s-0031-1291491 PMC3367502

[8] Islam RS Patel NC Lam-Himlin D Nguyen CC. Gastric polyps: a review of clinical, endoscopic, and histopathologic features and management decisions. Gastroenterol Hepatol (N Y) 2013;9:640–51. 24764778 PMC3992058

[9] Nagtegaal ID Odze RD Klimstra D. The 2019 WHO classification of tumours of the digestive system. Histopathology 2019;76 (2):182–8. 31433515 10.1111/his.13975 PMC7003895

[10] Elster K Morson BC. Histologic classification of gastric polyps. Pathology of the gastro-intestinal tract. Curr Top Pathol 1976;78–92. 10.1007/978-3-642-66481-6_3 795617

[11] Min C-C Wu J Hou F. Gastric pyloric gland adenoma resembling a submucosal tumor: a case report. World J Clin Cases 2020;8:2380–6. 32548171 10.12998/wjcc.v8.i11.2380 PMC7281057

[12] Çakar E Bayrak S Pasaoglu E Çolak S Bektas H Güneyi A. Pyloric gland adenoma: a case report. Case Rep Gastroenterol 2013;7:392–5. 24163651 10.1159/000355342 PMC3806671

[13] Schaefer I-M Cameron S Middel P. Pyloric gland adenoma of the cystic duct with malignant transformation: report of a case with a review of the literature. BMC Cancer 2012;12:10.1186/1471-2407-12-570 PMC3532145 23206236

[14] Vieth M Kushima R Borchard F Stolte M. Pyloric gland adenoma: a clinico-pathological analysis of 90 cases. Virchows Archiv 2003;442 (4):317–21. 12715167 10.1007/s00428-002-0750-6

[15] Golger D Probst A Wagner T Messmann H. Pyloric-gland adenoma of the stomach: case report of a rare tumor successfully treated with endoscopic submucosal dissection. Endoscopy 2008;40 (S 02):E110–1. 19085711 10.1055/s-2007-995554

[16] Salem SB Aadam AA Shapiro DM. Pyloric gland adenoma observed by magnifying endoscopy with narrow-band imaging. Dig Endosc 2014;26:754–5. 25115548 10.1111/den.12344

[17] Pei Q Shi Y Jing H. Early adenocarcinoma colliding with a pyloric gland adenoma in the gastric cardia. Gastrointest Endosc 2019;90:522–3. e1. 31022395 10.1016/j.gie.2019.04.225

[18] Kushima R Vieth M Borchard F Stolte M Mukaisho K-i Hattori T. Gastric-type well-differentiated adenocarcinoma and pyloric gland adenoma of the stomach. Gastric Cancer 2006;9:177–84. 16952035 10.1007/s10120-006-0381-8

[19] Vieth M Kushima R Mukaisho K-i Sakai R Kasami T Hattori T. Immunohistochemical analysis of pyloric gland adenomas using a series of Mucin 2, Mucin 5AC, Mucin 6, CD10, Ki67 and p53. Virchows Archiv 2010;457:529–36. 20827489 10.1007/s00428-010-0968-7

[20] Setia N Wanjari P Yassan L. Next-generation sequencing identifies 2 genomically distinct groups among pyloric gland adenomas. Hum Pathol 2020;97:103–11. 31783043 10.1016/j.humpath.2019.11.004

